# Carotid atherosclerosis progression in patients with Parkinson’s disease: a prospective cohort study

**DOI:** 10.3389/fnagi.2026.1928073

**Published:** 2026-09-30

**Authors:** Siyu Wang, Ni Yang, Haoyang Cheng, Qingbo Zhou

**Affiliations:** 1 Department of Neurology, The Second Qilu Hospital of Shandong University, Jinan, Shandong, China; 2 Department of Geriatric Medicine, Affiliated Hospital of Shandong University of Traditional Chinese Medicine, Jinan, China

**Keywords:** arterial plaque, carotid atherosclerosis, Parkinson’s disease, prospective cohort study, vascular protective phenotype

## Abstract

**Background:**

Evidence regarding the association between Parkinson’s disease (PD) and cardio-cerebrovascular risk remains conflicting, and it is unclear whether the dynamic progression of carotid atherosclerosis in PD patients differs from that in non-PD individuals. This study aimed to compare the progression of carotid atherosclerosis over a 1-year follow-up between PD patients and matched controls, and to investigate whether PD influences this progression independently of traditional cardiovascular risk factors.

**Methods:**

This prospective cohort study ultimately included 61 PD patients and 59 age- and sex-matched non-PD controls. Carotid ultrasound was performed at baseline and at the 1-year follow-up. The primary outcome was the annual change in total plaque area (ΔTPA), with plaque progression defined as ΔTPA ≥ 0.05 cm^2^. Multivariable logistic regression models, adjusting for diabetes, smoking, total cholesterol, and low-density lipoprotein cholesterol (LDL-C), were used to assess the independent association between PD status and plaque progression.

**Results:**

At baseline, carotid intima-media thickness and TPA were significantly lower in the PD group than in the control group (both *P* < 0.05). After 1 year, ΔTPA was significantly smaller in the PD group (*P* < 0.001), and the incidence of plaque progression was 6.56% in PD patients versus 25.42% in controls. Multivariable regression analysis showed that PD status was a protective factor against atherosclerosis progression (adjusted OR = 0.27, 95% CI: 0.074–0.984, *P* = 0.047). Within the PD group, no significant associations were observed between plaque progression and disease-specific factors such as disease duration, motor severity, or levodopa equivalent daily dose.

**Conclusion:**

Carotid atherosclerosis progression is significantly slower in PD patients than in non-PD individuals, and this effect is independent of traditional cardiovascular risk factors. These findings suggest that PD patients may exhibit a “vascular protective phenotype,” and that a unique shared regulatory network may exist between neurodegenerative processes and peripheral vascular homeostasis.

## Introduction

Parkinson’s disease (PD) is a neurodegenerative disorder characterized by progressive degeneration of dopaminergic neurons in the substantia nigra, with abnormal α-synuclein aggregation and subsequent mitochondrial dysfunction, oxidative stress, and neuroinflammation considered central to its pathology (Vázquez-Vélez and Zoghbi, 2021). However, recent evidence suggests that vascular health is a non-negligible concern in PD patients. A meta-analysis involving 101,712 PD patients demonstrated that the risk of stroke was significantly elevated by approximately 49% compared with non-PD individuals (OR: 1.49; 95% CI: 1.30–1.72) (Hu and Xu, 2023). In addition, epidemiological evidence suggests that the prevalence of traditional vascular risk factors, such as hypertension and diabetes, is higher among people with Parkinson’s disease; however, the conclusions of existing studies are not entirely consistent (Chohan et al., 2021; McLean et al., 2017). Reports of hypotension in patients with Parkinson’s disease typically describe the coexistence of orthostatic hypotension and supine hypertension, accompanied by significant blood pressure fluctuations, reflecting the complexity of vascular health issues in patients with Parkinson’s disease. These findings indicate that the comorbidity of PD and vascular disease warrants particular attention in clinical practice.

However, some clinical studies have reported opposite findings. As early as 1990, Struck et al. (1990) demonstrated in a case-control study that the cumulative incidence of ischemic stroke was significantly lower in PD patients than in age-matched controls, with a similar trend observed for myocardial infarction. The authors attributed this phenomenon to less tobacco use and milder systemic atherosclerosis in PD patients, and hypothesized that altered dopaminergic function might confer additional cerebrovascular protection (Struck et al., 1990). Recent studies have also identified inverse associations between certain traditional cardiovascular risk factors (e.g., smoking, high cholesterol) and the risk of developing PD (Vikdahl et al., 2015). An analysis of the ARIC cohort with up to 30 years of follow-up confirmed that midlife smoking was significantly inversely associated with the incidence of PD and PD dementia (HR: 0.36, 95% CI: 0.21–0.61) (Johri et al., 2025). These epidemiological associations suggest that PD patients may have a lower burden of traditional vascular risk factors; however, it cannot yet be directly inferred that they possess a vasoprotective phenotype. Therefore, it is necessary to conduct prospective studies using the intermediate phenotype of atherosclerotic progression.

Carotid intima-media thickness (CIMT) is widely recognized as a reliable indicator of systemic atherosclerotic burden and a strong predictor of cardio-cerebrovascular events (Zyriax et al., 2021). However, cross-sectional findings on CIMT in PD patients are inconsistent. Some studies have reported no significant difference in CIMT between PD patients and controls (Alves et al., 2021), whereas others have observed a positive correlation between increased CIMT and cognitive decline or disease severity within PD patients (Li et al., 2025). To date, prospective studies directly comparing the rate of atherosclerosis progression between PD patients and non-PD populations remain scarce.

In summary, the existing literature remains inconclusive regarding the association between Parkinson’s disease and atherosclerosis progression. On the one hand, PD patients have been reported to have an elevated stroke risk; on the other hand, evidence suggests that they exhibit less severe atherosclerosis, slower progression, and a “favorable” lipid profile. This discrepancy may stem from differences in study design, inadequate control of confounders, and selection bias. Therefore, rigorously designed prospective cohort studies are needed to directly compare the rate of atherosclerosis progression between PD patients and non-PD controls and to validate these observations with adequate adjustment for confounding factors.

This study aimed to systematically compare changes in atherosclerotic burden from baseline to 1-year follow-up between PD patients and age- and sex-matched non-PD controls in a prospective observational cohort study, using total plaque area (TPA) as the primary measure. Additionally, we analyzed the relationship between PD-specific characteristics and atherosclerosis progression, and explored the metabolic and clinical factors that may modulate this association. This study will help clarify the complex relationship between PD and vascular disease and provide evidence-based guidance for vascular risk management in PD patients.

## Materials and methods

### General information

This was a prospective observational cohort study conducted at Qilu Second Hospital of Shandong University between December 2024 and April 2026. The study protocol was approved by the Ethics Review Committee of Qilu Second Hospital of Shandong University (approval number: KYLL2025380), and written informed consent was obtained from all participants.

### Sample size considerations

As no prior studies have reported the incidence of plaque progression in PD patients specifically, a formal *a priori* sample size calculation for the binary outcome of plaque progression was not performed. The study was designed to detect a difference in the primary continuous endpoint, the annual change in total plaque area (ΔTPA), between PD patients and controls. A *post hoc* power calculation based on the observed effect size indicated that the primary analysis achieved >80% power. For the binary outcome of plaque progression, we acknowledge that the event rate was lower than expected, particularly in the PD group, and therefore analyses involving this outcome should be considered exploratory.

Parkinson’s disease patients were diagnosed according to the International Parkinson and Movement Disorder Society clinical diagnostic criteria and were recruited from neurology outpatient clinics and wards of the hospital. Exclusion criteria were: (1) secondary parkinsonism; (2) prior carotid artery stenting or endarterectomy; (3) severe heart failure, chronic kidney disease, or malignancy; (4) other neurodegenerative disorders (e.g., multiple system atrophy, progressive supranuclear palsy); and (5) inability to complete the 12-month follow-up. Control participants were community residents without PD or other parkinsonian syndromes who underwent routine health examinations at the Physical Examination Center of Qilu Second Hospital of Shandong University, and were matched to PD patients by age (±5 years) and sex. All included participants completed at least 12 months of follow-up.

### Baseline data

At baseline, demographic and clinical characteristics including age, sex, body mass index (BMI), smoking status (current or former smoker vs. never smoker), alcohol consumption (yes vs. no), and medical history (hypertension, diabetes mellitus, and coronary heart disease) were recorded for all participants. For PD patients, detailed medication information was collected, and the levodopa equivalent daily dose (LEDD) was calculated.

### Carotid ultrasound examination

At baseline and at the 1-year follow-up, a certified sonographer performed dual-energy ultrasound examinations of the carotid arteries using a Philips EPIQ 7C ultrasound system equipped with a 3–12 MHz linear array transducer and a 1–5 MHz curved array transducer. All carotid ultrasound examinations were performed by the same experienced sonographer, who was blinded to the participants’ group assignment (PD or control) and clinical information. The examinations were conducted in strict accordance with standard protocols for cervical artery ultrasound. The following three parameters were measured at both time points: (1) Carotid intima-media thickness (CIMT): measured bilaterally at the far wall of the common carotid artery 1 cm proximal to the bifurcation, the carotid bulb, and the internal carotid artery 1 cm distal to the bifurcation. The mean CIMT of all measurement sites from both sides was used for analysis (Saba et al., 2024; Zhou et al., 2021). (2) Total plaque area (TPA): longitudinal views that optimally displayed the largest cross-section of each plaque were obtained in the bilateral common carotid arteries, carotid bulbs, and proximal internal carotid arteries. Using the built-in measurement software, the boundary of each plaque was traced point by point to obtain its cross-sectional area (cm^2^). The sum of the cross-sectional areas of all visible plaques was calculated as the total plaque area. Archived carotid ultrasound images from 30 randomly selected participants at baseline and follow-up were selected. A second, uninformed operator independently performed the measurements to assess inter-operator reproducibility (ICC). A plaque was defined as a focal structure protruding into the lumen with a thickness of ≥0.5 mm or exceeding 50% of the surrounding intima-media thickness, or a thickness > 1.5 mm. The annual change for each parameter was calculated as the 1-year value minus the baseline value (Δ). The primary outcome was the annual change in total plaque area (ΔTPA). Plaque progression was defined as ΔTPA ≥ 0.05 cm^2^ (Spence et al., 2002).

### Laboratory tests

Fasting venous blood samples were collected at baseline and at the 1-year follow-up. At the Central Laboratory of Qilu Second Hospital, Shandong University, serum total cholesterol (TC), triglycerides (TG), low-density lipoprotein cholesterol (LDL-C), high-density lipoprotein cholesterol (HDL-C), blood glucose levels, neutrophil count, and lymphocyte count were measured using standard automated methods. The NLR was defined as the ratio of neutrophils to lymphocytes.

### Clinical and neuropsychological assessments

For PD patients, disease severity was assessed at baseline using the Unified Parkinson’s Disease Rating Scale (UPDRS) Part III and the Hoehn and Yahr (H-Y) stage. Cognitive function was evaluated with the Montreal Cognitive Assessment (MoCA). Non-motor symptoms were quantified using the Hamilton Anxiety Rating Scale (HAMA), the Hamilton Depression Rating Scale (HAMD), the Parkinson Fatigue Scale (PFS-16), and the Parkinson’s Disease Sleep Scale (PDSS). All assessments were performed by trained neurologists.

### Statistical analysis

All analyses were performed using SPSS version 25.0 and R version 4.3.1. Continuous variables are presented as mean ± SD or median (IQR) and compared using the *t*-test or Mann–Whitney U test as appropriate; categorical variables are presented as *n* (%) and compared using the Chi-square or Fisher’s exact test. The primary outcome was ΔTPA (1-year value minus baseline), compared between groups using the Mann–Whitney U test. A *post hoc* power calculation based on the observed ΔTPA effect size indicated >80% power. Plaque progression was defined as ΔTPA ≥ 0.05 cm^2^. Predictors of plaque progression were identified using univariate logistic regression; variables with *P* < 0.1 were included in the multivariate model, which comprised PD status, diabetes, smoking, total cholesterol, and LDL-C. The Hosmer–Lemeshow test was used to assess the model’s calibration. Given the limited number of events, although *P*-values were >0.1 in the univariate analysis, BMI and high-density lipoprotein cholesterol (HDL-C) were included as additional adjusters due to their known prognostic value and significant differences between groups. Additionally, to enhance comparability between the two groups, adjustments were made for baseline carotid intima-media thickness (CIMT) and baseline tissue-type plasminogen activator (TPA), respectively. Within the PD group, univariable logistic regression was used to explore associations between disease-specific characteristics and plaque progression, including disease duration, LEDD, H-Y stage, MoCA, UPDRS Part III, HAMA, HAMD, PFS-16, PDSS, and motor subtype. All tests were two-sided; *P* < 0.05 was considered statistically significant.

## Results

### Participant baseline characteristics

Initially, 140 participants were enrolled, comprising 74 patients with Parkinson’s disease (PD) and 66 non-PD controls. During the 1-year follow-up, 17 participants were lost to follow-up (11 in the PD group and six in the control group), and an additional three were excluded due to missing key data, such as carotid ultrasound measurements. We also performed a baseline data analysis of participants ultimately included in the study and those lost to follow-up, and there was no statistically significant difference between the two groups (Supplementary Tables 2, 3). Thus, the final analytical sample comprised 120 participants (61 PD patients and 59 controls). Baseline characteristics of the two groups are summarized in Table 1. There were no statistically significant differences between the two groups in the use of glucose-lowering, antihypertensive, antithrombotic, and lipid-lowering medications (Supplementary Table 1).

**TABLE 1:** Baseline characteristics of the Parkinson’s disease group and the control group.

Variable	PD group (*n* = 61)	Control group (*n* = 59)	*P*-value
Gender (male)	30 (49.18%)	35 (59.32%)	0.265
Age (year)	66.44 ± 8.88	66.97 ± 8.87	0.742
BMI (kg/m^2^)	22.96 ± 2.34	24.22 ± 2.96	0.011
Hypertension (yes)	23 (37.7%)	29 (49.15%)	0.206
Diabetes (yes)	15 (24.6%)	15 (25.4%)	0.916
Smoke (yes)	14 (23%)	18 (30.51%)	0.351
Drink (yes)	25 (40.98%)	18 (30.51%)	0.349
Blood sugar (mmol/L)	5.59 (4.99, 6.44)	5.9 (5.06, 7.2)	0.308
TG (mmol/L)	0.95 (0.75, 1.3)	1.13 (0.92, 1.67)	0.012
TC (mmol/L)	3.69 ± 1.46	4.3 ± 1.09	0.015
HDL-C (mmol/L)	1.36 (1.09, 1.97)	1.15 (0.93, 1.34)	<0.001
LDL-C (mmol/L)	1.68 (1.24, 2.39)	2.44 (1.73, 2.88)	0.002
NLR	2.32 (1.61, 3.13)	2.45 (1.64, 3.8)	0.439
CIMT	0.9 (0.8, 1.0)	0.8 (0.75, 0.85)	<0.001
TPA	0.24 (0.07, 0.55)	0.56 (0.17, 1.01)	0.003

Continuous variables with a normal distribution are expressed as mean ± standard deviation, and *P*-values were calculated using the independent samples *t*-test. Continuous variables with a non-normal distribution are expressed as median (interquartile range); comparisons between groups were performed using the Mann-Whitney U test. Categorical variables are expressed as percentages; *P*-values were calculated using the weighted chi-square test. BMI, body mass index; TG, triglycerides; TC, total cholesterol; LDL-C, low-density lipoprotein cholesterol; HDL-C, high-density lipoprotein cholesterol; NLR, Neutrophil-to-Lymphocyte Ratio; CIMT, carotid intima-media thickness; TPA, total plaque area.

Compared with controls, PD patients had significantly lower BMI (*P* = 0.011), triglycerides (*P* = 0.012), total cholesterol (*P* = 0.015), and LDL-C (*P* = 0.002), while HDL-C was significantly higher (*P* < 0.001). Regarding vascular parameters, both baseline CIMT (*P* < 0.001) and baseline TPA (*P* = 0.003) were significantly lower in the PD group. There were no significant differences in age, sex, fasting blood glucose, prevalence of hypertension, prevalence of diabetes, smoking status, or alcohol consumption between the two groups (all *P* > 0.05).

### Changes in carotid ultrasound parameters over 1 year

Changes in carotid ultrasound parameters over the 1-year period are summarized in Table 2. For the primary endpoint, ΔTPA was significantly smaller in the PD group than in the control group (*P* < 0.001). As a prespecified exploratory analysis, the magnitude of ΔCIMT was also significantly lower in the PD group, consistent with the direction of the primary endpoint. The inter-rater reliability coefficient (ICC) for the ΔTPA assay was 0.97 (95% confidence interval: 0.94–0.99), indicating good reproducibility.

**TABLE 2:** Comparison of changes in CIMT and TPA between the Parkinson’s disease (PD) group and the control group over 1 year.

Variable	PD group	Control group	*P*-value
△CIMT	0.05 (0, 0.1)	0.1 (0.05, 0.15)	0.002
△TPA	0.02 (0.01,0.02)	0.04 (0.03,0.045)	<0.001
Plaque progression, *n* (%)	4 (6.56%)	15 (25.42%)	<0.001

Continuous variables with a non-normal distribution are expressed as median (interquartile range); comparisons between groups were performed using the Mann-Whitney U test. △CIMT, change in carotid intima-media thickness over 1 year; △TPA, change in total plaque area over 1 year.

### PD status and atherosclerosis progression

During the 1-year follow-up, plaque progression (defined as ΔTPA ≥ 0.05 cm^2^) occurred in four PD patients (6.56%) and 15 controls (25.42%). Univariable logistic regression identified the following candidate predictors with *P* < 0.1: PD status (OR = 0.206, 95% CI: 0.064–0.664, *P* = 0.008), diabetes (OR = 3.429, 95% CI: 1.235–9.515, *P* = 0.018), smoking (OR = 3.052, 95% CI: 1.108–8.41, *P* = 0.031), total cholesterol (OR = 1.639, 95% CI: 1.074–2.502, *P* = 0.022), and LDL-C (OR = 2.406, 95% CI: 1.351–4.286, *P* = 0.003) (Table 3). In the multivariable logistic regression model adjusting for diabetes, smoking, total cholesterol, and LDL-C, PD status remained significantly associated with a lower risk of atherosclerosis progression (OR = 0.27, 95% CI: 0.074–0.984, *P* = 0.047) (Table 4). We have reported the Hosmer-Lemeshow goodness-of-fit test for the multivariable model (*P* = 0.684), indicating that the model fit was acceptable. Since BMI and HDL-C are known predictors of atherosclerosis and showed significant between-group differences, and sex also influences atherosclerosis progression, we additionally included them in the multivariable logistic regression analysis despite *P* > 0.1 in univariable analysis. The PD group had significantly lower plaque burden and CIMT at baseline, which complicates the interpretation of change measures. To determine whether the observed difference was attributable to the lower initial plaque burden, we performed sensitivity analyses by separately adding baseline CIMT and TPA to the multivariable logistic regression models. These results are presented in Table 5. We obtained complete cases through multiple imputation and performed a sensitivity analysis; the results remained significant (Supplementary Table 4).

**TABLE 3:** Univariate logistic regression analysis of factors associated with carotid plaque progression.

Variable	OR (95% CI)	*P*
PD	0.206 (0.064, 0.664)	0.008
Gender	0.644 (0.234, 1.77)	0.394
Age	1.011 (0.954, 1.07)	0.719
BMI	1.109 (0.931, 1.32)	0.246
Hypertension	2.012 (0.745, 5.435)	0.168
Diabetes	3.429 (1.235, 9.515)	0.018
Smoke	3.052 (1.108, 8.410)	0.031
Drink	1.371 (0.505, 3.723)	0.535
Blood sugar	1.114 (0.915, 1.355)	0.282
TG	1.054 (0.446, 2.495)	0.904
TC	1.639 (1.074, 2.502)	0.022
LDL-C	2.406 (1.351, 4.286)	0.003
HDL-C	0.681 (0.265, 1.753)	0.426
NLR	0.913 (0.69, 1.209)	0.525

BMI, body mass index; TG, triglycerides; TC, total cholesterol; LDL-C, low-density lipoprotein cholesterol; HDL-C, high-density lipoprotein cholesterol; NLR, neutrophil-to-lymphocyte ratio.

**TABLE 4:** Multivariable logistic regression analysis of factors associated with carotid plaque progression.

Variable	OR (95% CI)	*P*
PD	0.27 (0.074, 0.984)	0.047
Diabetes	3.866 (1.216, 12.289)	0.022
Smoke	2.697 (0.863, 8.428)	0.088
TC	1.227 (0.712, 2.112)	0.461
LDL-C	1.981 (1.019, 3.849)	0.044
Hosmer-Lemeshow test	–	0.684

PD, Parkinson disease; TC, total cholesterol; LDL-C, low-density lipoprotein cholesterol.

**TABLE 5:** Multivariable logistic regression analysis of factors associated with carotid plaque progression.

Model	Covariates	OR (95% CI)	*P*
Primary	PD + diabetes + smoking + TC + LDL-C	0.27 (0.074, 0.984)	0.047
Sensitivity 1	Primary + BMI	0.314 (0.083, 1.191)	0.089
Sensitivity 2	Primary + HDL-C	0.191 (0.083, 0.93)	0.040
Sensitivity 3	Primary + BMI + HDL-C	0.224 (0.046, 1.100)	0.065
Sensitivity 4	Primary + gender	0.269 (0.073, 0.987)	0.048
Sensitivity 5	Primary + baseline CIMT	0.259 (0.068, 0.99)	0.048
Sensitivity 6	Primary + baseline TPA	0.646 (0.036, 11.721)	0.764

PD, Parkinson disease; TC, total cholesterol; LDL-C, low-density lipoprotein cholesterol; CIMT, carotid intima-media thickness; TPA, total plaque area.

### Association between disease-specific factors and atherosclerosis progression in PD patients

Among the 61 PD patients, univariable logistic regression was used to examine associations between disease-specific characteristics and plaque progression (ΔTPA ≥ 0.05 cm^2^). The variables analyzed included H-Y stage, MoCA score, UPDRS Part III score, HAMA score, HAMD score, PFS-16 score, PDSS score, and motor subtype (tremor-dominant vs. others). None of these factors reached statistical significance (all *P* > 0.05) (Table 6).

**TABLE 6:** Univariable logistic regression analysis of disease-specific factors associated with carotid plaque progression in PD patients.

Variable	OR (95% CI)	*P*
MoCA	1.002 (0.872, 1.152)	0.977
H-Y stage	0.667 (0.065, 6.847)	0.733
UPDRS 3	0.979 (0.918, 1.045)	0.523
Motor subtype	0.393 (0.081, 1.899)	0.245
HAMA	1.025 (0.908, 1.148)	0.686
HAMD	1.004 (0.893, 1.128)	0.947
PFS-16	1.004 (0.947, 1.065)	0.892
PDSS	1.002 (0.951, 1.056)	0.938
Disease duration	0.958 (0.525, 1.746)	0.888
LEDD	0.973 (0.944, 1.002)	0.072

MoCA, Montreal Cognitive Assessment; H-Y stage, Hoehn and Yahr stage; UPDRS Part III, Unified Parkinson’s Disease Rating Scale Part III; Motor subtype, tremor-dominant versus others (including akinetic-rigid, mixed, and postural instability/gait difficulty); HAMA, Hamilton Anxiety Rating Scale; HAMD, Hamilton Depression Rating Scale; PFS-16, Parkinson Fatigue Scale-16; PDSS, Parkinson’s Disease Sleep Scale; LEDD, levodopa equivalent daily dose.

## Discussion

This 1-year prospective cohort study found that, compared with non-PD controls, PD patients exhibited thinner baseline carotid intima-media thickness, smaller total plaque area, and significantly slower progression of total plaque burden during follow-up. After adjusting for traditional cardiovascular risk factors including diabetes, smoking, total cholesterol, and low-density lipoprotein cholesterol, PD status remained independently associated with an approximately 73% lower risk of atherosclerosis progression (OR = 0.27, 95% CI: 0.074–0.984). After adjusting for known cardiovascular risk factors, the presence of PD remained independently associated with a slower progression of atherosclerosis; therefore, we believe that patients with PD may possess a unique “vascular protective phenotype.” After adjusting for HDL-C, PD status remained independently associated with plaque progression (*P* = 0.040), confirming the robustness of the results. After adjusting for BMI, PD status still tended to be inversely associated with plaque progression (OR = 0.31, 95% CI: 0.083–1.191), although statistical significance was not reached; simultaneous adjustment for both further attenuated the association, possibly reflecting collinearity and limited sample size. Because the two groups differed significantly in baseline plaque burden and CIMT, we performed sensitivity analyses by separately adding baseline CIMT and TPA to the multivariable logistic regression models. When baseline CIMT was included, PD status still independently influenced carotid atherosclerosis progression; however, after adjusting for baseline TPA, the association between PD status and atherosclerosis progression was no longer statistically significant. We consider that the vascular protective phenotype in PD is mainly reflected by a lower baseline plaque burden and the consequent slower progression, which represent a continuous process. Within the PD group, however, disease duration, motor severity, cognitive status, non-motor symptom burden, and motor subtype showed no significant association with atherosclerosis progression. These findings provide new insights into the relationship between Parkinson’s disease and atherosclerosis.

In this study, baseline CIMT and total plaque area were significantly lower in the PD group than in controls, consistent with the hypothesis proposed by Struck et al. (1990) that PD patients have a lower atherosclerotic burden. Recent epidemiological evidence also suggests that genetic susceptibility to PD is associated with lower LDL-C levels and reduced risk of coronary heart disease, reflecting a seemingly “favorable” cardiovascular metabolic profile (Fang et al., 2019; Guo et al., 2015; Lv et al., 2022). Our study not only confirmed lower TC and LDL-C levels and higher HDL-C levels in PD patients, but also demonstrated through prospective follow-up that this metabolic phenotype translates into attenuated atherosclerosis progression in a real-world setting. This protective association remained significant after statistical adjustment, indicating that it is not fully explained by between-group differences in traditional risk factors and that deeper biological mechanisms may underlie this phenomenon.

Regarding potential mechanisms underlying the slower atherosclerosis progression in PD patients, the direct interaction between the dopaminergic system and the peripheral vasculature should be considered first (Osinga et al., 2017). Dopamine receptors are widely expressed in vascular smooth muscle cells and endothelial cells, and dopaminergic signaling can exert vasodilatory and antiproliferative effects by inhibiting endothelin-1 release, modulating the renin-angiotensin-aldosterone system, and suppressing oxidative stress (Basu et al., 2001; Sarkar et al., 2017; Yang et al., 2021). Although central dopamine is severely depleted in PD, compensatory changes in peripheral dopaminergic activity and systemic exposure to levodopa therapy may produce a complex net effect on the vessel wall. On the one hand, dopamine may confer protection by improving microvascular structure and neurovascular coupling (Hou et al., 2026); on the other hand, under certain conditions (e.g., long-term high-dose treatment, presence of vascular risk factors), it may exacerbate vascular inflammation, aberrant angiogenesis, or endothelial dysfunction, thereby influencing disease progression and treatment response (Booth et al., 2021; Kim et al., 2023). Although this study found no association between levodopa equivalent daily dose and atherosclerosis progression, given the limitations of sample size and follow-up duration, a long-term modulatory effect of the medication on vascular wall inflammation and endothelial function cannot be ruled out. The significantly slower progression of total plaque area in PD patients in this study suggests that the growth of atherosclerotic burden is suppressed, which may directly translate into a reduced risk of long-term cerebrovascular events. This finding may help reconcile the discrepancy between the elevated stroke risk in PD patients reported by the aforementioned meta-analysis and the slower subclinical atherosclerosis progression observed in this study (Hu and Xu, 2023). Although the risk of stroke events is increased, the primary driver may not be large artery atherosclerotic burden, but rather factors such as increased blood pressure variability due to autonomic dysfunction, higher risk of atrial fibrillation, or differences in anticoagulation management.

Beyond pharmacological factors, the pathophysiological features of PD itself may also influence vascular status. Abnormal aggregation of α-synuclein occurs not only in the central nervous system but may also involve peripheral tissues and platelets. Studies have shown that platelet function is altered in PD patients, with reduced aggregation capacity (Kumar Beura et al., 2024; Tirozzi et al., 2020). Although meta-analyses indicate an increased risk of stroke in patients with Parkinson’s disease (PD), this risk may be driven primarily by non-atherosclerotic mechanisms, such as blood pressure fluctuations caused by autonomic dysfunction, an increased risk of atrial fibrillation, and differences in anticoagulation management; conversely, impaired platelet function in PD patients may reduce arterial thrombosis, which could partially explain why, despite slower progression of atherosclerosis, their overall risk of stroke remains high. Furthermore, PD-related oxidative stress and neuroinflammation may affect vascular endothelial function through complex systemic interactions (Förstermann et al., 2017; Trist et al., 2019).

Several alternative explanations warrant consideration regarding the observation that atherosclerosis progresses more slowly in patients with Parkinson’s disease (PD). At baseline, we observed a more favorable lipid profile in PD patients. To determine whether differences in lipid metabolism levels were the cause of the differences in disease progression, we adjusted for total cholesterol and low-density lipoprotein cholesterol in our multivariate models; the results still showed statistically significant differences, suggesting that the slower progression was not solely attributable to the measured lipid levels. Additionally, survival bias may have contributed to the observed association. If individuals with more severe atherosclerosis are more likely to die before developing or being diagnosed with Parkinson’s disease, then the Parkinson’s disease group would consist of a higher proportion of survivors with a lighter atherosclerotic burden. However, based on a 1-year follow-up of vascular stiffness, the rate of change in CIMT and TPA in the PD group was slower than that in the control group, suggesting that survival bias cannot fully account for this finding. Another possible mechanism is that reduced physical activity in people with Parkinson’s disease may secondarily affect lipid metabolism and vascular health; however, such confounding factors typically lead to worsening vascular status—since physical inactivity is a known cardiovascular risk factor—and therefore are unlikely to be the cause of the observed slowing of disease progression. In summary, the study design and statistical adjustments rule out the simplest explanations for the observed lipid differences; however, survival bias and unmeasured lifestyle factors remain unresolved and should be addressed in future studies with larger sample sizes, longer follow-up periods, and objective monitoring of physical activity.

The findings of this study have important implications for the clinical management of PD patients. Although PD patients appear to have slower atherosclerosis progression and a more favorable lipid profile, clinicians should remain vigilant regarding the potentially elevated risk of stroke, and should particularly emphasize screening and management of circadian blood pressure rhythm, orthostatic hypotension, and atrial fibrillation. It is also inadvisable to completely abandon primary prevention with statins based solely on low LDL-C levels in PD patients, given the complex balance between atherosclerosis regression and plaque stabilization; the benefit threshold of lipid-lowering therapy in elderly populations with neurodegenerative diseases requires individualized assessment.

Several limitations of this study must be acknowledged. First, the sample size was relatively small and the follow-up period was only 1 year. Second, as an observational study, although key confounders were adjusted for, residual confounding (e.g., physical activity, dietary habits, and psychosocial factors) and potential reverse causality cannot be completely excluded. Third, imaging markers of cerebral small vessel disease, such as enlarged perivascular spaces, cerebral microbleeds, and white matter hyperintensities, were not incorporated. Furthermore, although we adjusted for major traditional risk factors, we cannot completely rule out the possibility of residual confounding due to survival bias and unmeasured lifestyle factors (such as physical activity and dietary habits). These factors may have contributed to some extent to the observed vasoprotective phenotype.

In summary, from the perspective of dynamic carotid atherosclerosis progression, this prospective cohort study provides initial evidence that PD patients exhibit a slower vascular aging phenotype over a 1-year follow-up period. This protective effect is independent of traditional cardiovascular risk factors, suggesting that a unique intrinsic regulatory network may exist between the neurodegenerative process of PD and peripheral vascular remodeling. Future studies with larger multicenter cohorts, integrating genetic, metabolomic, and vascular function assessments, are warranted to further elucidate the precise interactions among dopaminergic signaling, α-synuclein pathology, and vascular homeostasis, thereby providing a scientific basis for more precise cerebrovascular disease prevention strategies in PD patients.

## Data Availability

The raw data supporting the conclusions of this article will be made available by the authors, without undue reservation.
