# Supplementary material for: Carotid atherosclerosis progression in patients with Parkinson’s disease: a prospective cohort study
Source: Front Aging Neurosci. 2026 Sep 30;18:1928073. doi: 10.3389/fnagi.2026.1928073 (PMC13662106; doi:10.3389/fnagi.2026.1928073)
Supplement: Supplementary file 1 [file Data_Sheet_1.docx]

**Supplementary Table 1. Medication Use in the PD Group and the control Group.**

| Types of Medications | PD group  （n=61） | control group（n=59） | P-value |
| --- | --- | --- | --- |
| Blood Pressure Medications |  |  | 0.280 |
| Yes | 22（44.9%） | 27（55.1%） |  |
| No | 39 (54.93%) | 32 (45.07%) |  |
| Diabetes Medications |  |  | 0.741 |
| Yes | 15 (53.57%) | 13 (46.43%) |  |
| No | 46 (50%) | 46 (50%) |  |
| Cholesterol-Lowering Medications |  |  | 0.664 |
| Yes | 10 (55.56%) | 8 (44.44%) |  |
| No | 51 (50%) | 51 (50%) |  |
| Antiplatelet Medications |  |  | 0.583 |
| Yes | 9 (60%) | 6 (40%) |  |
| No | 52 (49.52%) | 53 (50.48%) |  |

Categorical variables are expressed as percentages; P-values were calculated using the weighted chi-square test.

**Supplementary Table 2. Baseline Characteristics of the Completion Group and the Lost-to-Follow-Up Group Among Patients with Parkinson's Disease.**

| **Variable** | Completion Group（n=61） | Loss-to-Follow-Up Group（n=11） | P-value |
| --- | --- | --- | --- |
| Gender（Male） | 30（49.18%） | 7（63.64%） | 0.564 |
| Age (year) | 66.44±8.88 | 64.82±9.02 | 0.579 |
| BMI (kg/m^2^) | 22.96±2.34 | 22.47±2.41 | 0.525 |
| Disease Duration（year） | 2（1, 3） | 2（1.5, 2.5） | 0.390 |
| LEDD | 477±257 | 489±212 | 0.894 |
| Hypertension（Yes） | 23（37.7%） | 5（45.4%） | 0.633 |
| Diabetes（Yes） | 15（24.6%） | 3（27.3%） | 0.422 |
| Smoke（Yes） | 14（23%） | 3（27.3%） | 0.351 |
| Drink（Yes） | 25（40.98%） | 5（45.4%） | 0.786 |
| NLR | 2.32（1.61, 3.13） | 1.96（1.47, 3.1） | 0.419 |
| Blood Glucose | 5.59（5, 4.44） | 5.36（4.66, 5.74） | 0.228 |
| TG (mmol/L) | 0.95（0.75, 1.3） | 1.1（0.9, 1.37） | 0.330 |
| TC (mmol/L) | 3.69±1.46 | 2.80±1.05 | 0.057 |
| HDL-C (mmol/L) | 1.36（1.09, 1.97） | 1.29（0.76, 1.56） | 0.484 |
| LDL-C (mmol/L) | 1.68（1.24, 2.39） | 1.68（1.14, 2.12） | 0.121 |
| MoCA | 23（15.5, 26） | 14（13, 22） | 0.105 |
| H-Y stage | 1（1, 2） | 1（1, 2） | 0.820 |
| UPDRSⅢ | 38±16.22 | 44.91±18.84 | 0.209 |
| motor subtype | 1（1, 3） | 2（1, 2） | 0.655 |
| HAMA | 17（12, 25.5） | 15（12, 22） | 0.492 |
| HAMD | 17（10, 21.5） | 17（10, 23） | 0.770 |
| PFS-16 | 37.36±17.46 | 34.91±11.95 | 0.657 |
| PDSS | 43.52±19.56 | 35.45±12.89 | 0.193 |
| CIMT | 0.9（0.8, 1.0） | 0.75（0.7, 0.9） | 0.429 |
| TPA | 0.24（0.07, 0.55） | 0.25（0.13, 0.66） | 0.785 |

Continuous variables with a normal distribution are expressed as mean ± standard deviation, and P-values were calculated using the independent samples t-test. Continuous variables with a non-normal distribution are expressed as median (interquartile range); comparisons between groups were performed using the Mann-Whitney U test. Categorical variables are expressed as percentages; P-values were calculated using the weighted chi-square test. Abbreviations: BMI, body mass index; LEDD, levodopa equivalent daily dose; NLR, Neutrophil-to-Lymphocyte Ratio. TG, triglycerides; TC, total cholesterol; LDL-C, low-density lipoprotein cholesterol; HDL-C, high-density lipoprotein cholesterol. MoCA, Montreal Cognitive Assessment; H-Y stage, Hoehn and Yahr stage; UPDRS Part III, Unified Parkinson’s Disease Rating Scale Part III; motor subtype, tremor-dominant versus others (including akinetic-rigid, mixed, and postural instability/gait difficulty); HAMA, Hamilton Anxiety Rating Scale; HAMD, Hamilton Depression Rating Scale; PFS-16, Parkinson Fatigue Scale-16; PDSS, Parkinson’s Disease Sleep Scale; CIMT, carotid intima-media thickness; TPA, total plaque area.

**Supplementary Table 3. Baseline Characteristics of the Completion Group and the Lost-to-Follow-Up Group in the Control Cohort.**

| **Variable** | Completion Group（n=59） | Loss-to-Follow-Up Group（n=9） | P-value |
| --- | --- | --- | --- |
| Gender（Male） | 34（57.63%） | 5（55.56%） | 0.908 |
| Age (year) | 66.97±8.87 | 64.78±5.55 | 0.208 |
| BMI (kg/m^2^) | 24.22±2.96 | 22.21±2.04 | 0.055 |
| Hypertension（Yes） | 30（50.85%） | 3（33.33%） | 0.331 |
| Diabetes（Yes） | 16（27.12%） | 2（22.22%） | 0.758 |
| Smoke（Yes） | 17（28.81%） | 3（33.33%） | 0.783 |
| Drink（Yes） | 19（32.2%） | 4（44.44%） | 0.473 |
| NLR | 2.45（1.64,3.8） | 3.22（1.83,4.61） | 0.405 |
| Blood Glucose | 5.9（5.06,7.2） | 5.1（4.86,5.78） | 0.105 |
| TG (mmol/L) | 1.13（0.92,1.67） | 1.41（1.09,2.64） | 0.069 |
| TC (mmol/L) | 4.29±1.1 | 4.54±0.8 | 0.505 |
| HDL-C (mmol/L) | 1.15（0.93,1.34） | 1.16（0.9,1.44） | 0.758 |
| LDL-C (mmol/L) | 2.44（1.67,2.9） | 2.52（1.87,3.39） | 0.4 |
| CIMT | 0.9（0.8,1） | 0.85（0.75,0.9） | 0.534 |
| TPA | 0.56（0.17,1.01） | 0.36（0.17,0.89） | 0.772 |

Continuous variables with a normal distribution are expressed as mean ± standard deviation, and P-values were calculated using the independent samples t-test. Continuous variables with a non-normal distribution are expressed as median (interquartile range); comparisons between groups were performed using the Mann-Whitney U test. Categorical variables are expressed as percentages; P-values were calculated using the weighted chi-square test. Abbreviations: BMI, body mass index; NLR, Neutrophil-to-Lymphocyte Ratio. TG, triglycerides; TC, total cholesterol; LDL-C, low-density lipoprotein cholesterol; HDL-C, high-density lipoprotein cholesterol; CIMT, carotid intima-media thickness; TPA, total plaque area.

**Supplementary Table 4：Complete-case vs Multiple-imputation sensitivity analyses.**

| Model | Covariates | OR (95% CI) | P |
| --- | --- | --- | --- |
| Primary | PD + diabetes + smoking + TC + LDL-C | 0.183（0.043,0.771) | 0.021 |
| Sensitivity 1 | Primary + BMI | 0.235（0.054,1.025) | 0.054 |
| Sensitivity 2 | Primary + HDL-C | 0.13（0.021,0.815) | 0.029 |
| Sensitivity 3 | Primary + BMI + HDL-C | 0.174（0.029,1.056) | 0.057 |
| Sensitivity 4 | Primary + Gender | 0.173 (0.039,0.756) | 0.020 |
| Sensitivity 5 | Primary + Baseline CIMT | 0.183 (0.042, 0.802) | 0.025 |
| Sensitivity 6 | Primary + Baseline TPA | 0.297 (0.026,3.354) | 0.323 |

Abbreviations: PD, Parkinson disease; TC, total cholesterol; LDL-C, low-density lipoprotein cholesterol; CIMT, carotid intima-media thickness; TPA, total plaque area.
